# Physical activity, sedentary behavior and functionality in older adults: A cross-sectional path analysis

**DOI:** 10.1371/journal.pone.0246275

**Published:** 2021-01-29

**Authors:** Cíntia Aparecida Garcia Meneguci, Joilson Meneguci, Jeffer Eidi Sasaki, Sheilla Tribess, Jair Sindra Virtuoso Júnior

**Affiliations:** 1 Graduate Program in Health Care, Federal University of Triangulo Mineiro (UFTM), Uberaba, MG, Brazil; 2 Graduate Program in Physical Education, Federal University of Triangulo Mineiro (UFTM), Uberaba, MG, Brazil; University of New South Wales, AUSTRALIA

## Abstract

Disability is negatively associated with the health of older adults, and it can be mediated by healthy lifestyles and behaviors throughout one’s life. In this context, understanding the interrelationships between sedentary behavior, physical activity and functionality may assist in the implementation of effective public health actions. Thus, the aim of the present study was to investigate the relationships between both physical activity and sedentary behavior and functionality in older adults and the possible mediators. The variables analyzed were selected according to the content analysis of International Classification of Functioning, Disability and Health model, and included activity, participation, health conditions, body functions and structures, environmental factors and personal factors. 419 individuals participated in the study. Physical activity was directly associated with disability in instrumental activities of daily living (IADL), and the association was mediated by self-esteem, aerobic endurance, and agility/balance. Sedentary behavior was indirectly associated with IADL disability, and the association was mediated by aerobic resistance, nutritional status, and agility/balance. Regarding the basic activities of daily living (BADL), physical activity showed an indirect association mediated by aerobic resistance and IADL. The association of sedentary behavior with BADL was mediated by aerobic resistance and lower limb flexibility. These results reinforce the idea that functionality is multidimensional, and the mediating factors must be considered when strategies for promoting physical activity and reducing sedentary behavior are designed.

## Introduction

Human aging is considered an inevitable process that is inherent to all structures and functions of the body, and it is considered an independent risk factor for the development and progression of non-communicable chronic diseases, such as cardiovascular disease, cancer, type 2 diabetes mellitus and dementia [1]. However, aging, even in the absence of chronic diseases, involves some functional loss, either physical or cognitive [2,3].

In this context, disability has become a public health concern due to the increasing demand and costs for long-term care [4]. Disability has been described by some authors as the inability to perform or difficulty in performing daily activities and tasks that are normally indispensable for a socially independent life [5].

From a broader viewpoint, according to the International Classification of Functioning, Disability and Health (ICF) model, disability is described as the result of relationships between a disease, limitations in activities and restrictions in social participation, in addition to the influences of personal and environmental factors that may act as barriers or facilitators in the performance of each task [6–8].

The ICF model consists of three dimensions that are interconnected and describe the health-related states. The first is the biomedical dimension, which corresponds to individual disease or health condition. The second dimension includes functionality and disability (body functions and structures, activity and participation) and the third inserts context factors (personal and environmental factors) [6–8].

The preservation of functionality in older adults requires the adoption of healthy lifestyles and behaviors throughout life [9]. Although the benefits of regular physical activity for the health of older adults are well known, there is a high prevalence of people aged 60 and over who do not perform physical activity regularly, which exacerbates physical and functional decline [10]. In addition, sedentary behavior is associated with disability regardless of whether an individual participates in moderate to vigorous physical activity [11,12].

The interrelationships between the components of the ICF theoretical model have previously been analyzed in adults with different health conditions in a rehabilitation center, with an average age of 42.0 (SD = 12.1) [13] and individuals with osteoarthritis with average age 68.9 (SD = 9.4) of [14]. However, the explanatory relationships between the ICF constructs in older adults and their relationships with physical activity and sedentary behavior remain unknown.

Therefore, it is clear that understanding the interrelationships between sedentary behavior, physical activity and disability may assist in the implementation of effective public health actions that may deter disability in older adults. Thus, the aim of the present study was to investigate the relationships between physical activity and sedentary behavior and functionality in older adults and the possible mediators.

## Materials and methods

This observational cross-sectional analytical study was conducted in the municipality of Alcobaça, which is located in the far southern region of the state of Bahia in Brazil. The study was evaluated and approved by the Ethics Committee on Research with Human Beings of the Federal University of Triangulo Mineiro—UFTM (No. 966.983/2015).

The study population included individuals aged 60 years or older who were living in urban areas and were registered in the Family Health Strategy, which is a program that aims to reorganize primary healthcare in the country, according to the precepts of the Brazilian Unified Health System. The exclusion criteria were as follows: being bedridden; being hospitalized; being a resident in long-term institutions; having severe difficulty in visual and hearing acuity that can make communication with the interviewer difficult; being wheelchair dependent; having musculoskeletal or neurological diseases that prevent physical function measurements from being taken; failing to score ≥13 points in the Mini Mental State Examination (MMSE) [15]; and not agreeing to participate in the study by signing the informed consent form.

Of the 743 individuals enrolled in the Family Health Strategy, 54 refused to participate in the research, 58 were excluded on the basis of the study criteria (six were wheelchair users; 10 were bedridden; 19 had a previous diagnosis of diseases or dysfunctions that made the interview impossible; 14 had an MMSE, eight had communication difficulties, and one was an alcoholic), 158 older adults could not be contacted after three attempts, and 54 did not have data for all the specific variables of the present study. Thus, data for 419 older adults were analyzed in this study.

The data were collected between July and October 2015. With the help of community health agents in the municipality, the eligible older adults were located and were informed of the research objectives. Those who agreed to participate signed the informed consent form, participated in a face-to-face interview to obtain sociodemographic (age, sex, years of education, family arrangement, marital status, and income), health and behavioral information, and underwent physical function tests. The interviewers were previously trained.

**Fig 1:**
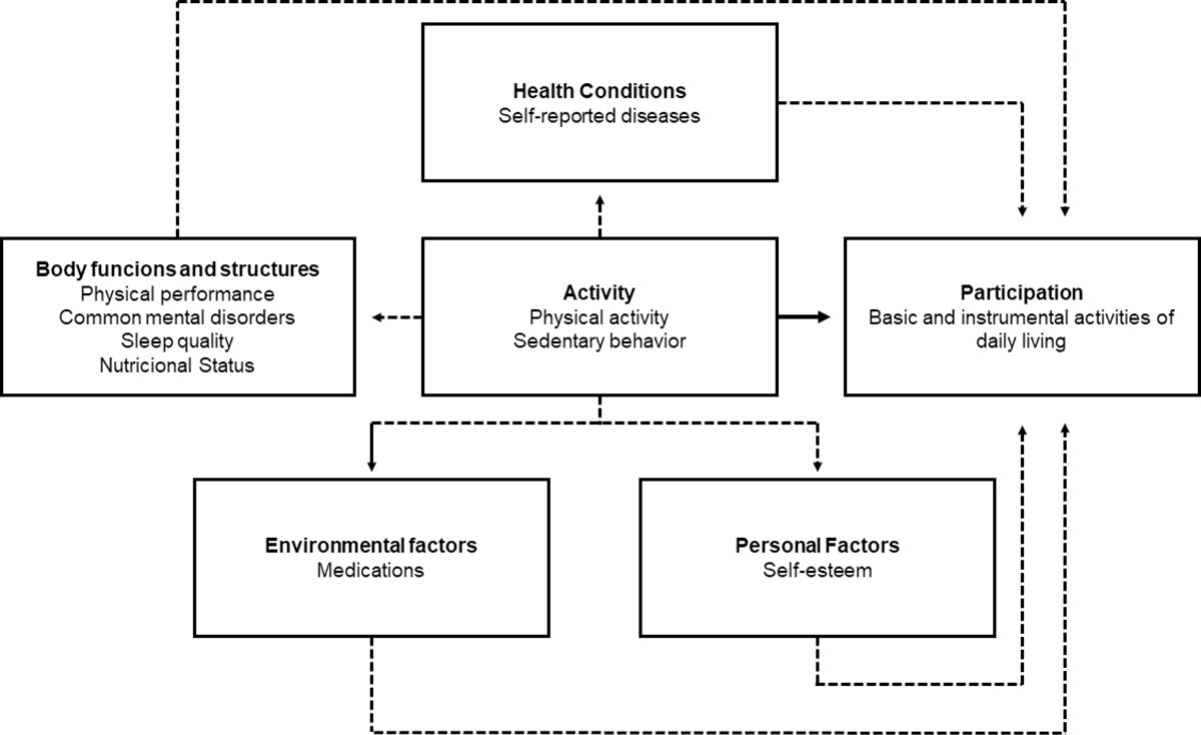
Variables tested according to the theoretical model of the international classification of functionality, disability and health. Note: Dotted lines refer to indirect effects of physical activity and sedentary behavior on functionality. Solid line is the direct effect of physical activity and sedentary behavior on functionality.

Considering the ICF model, the variables tested in present study were: physical activity and sedentary behavior (activity), performing instrumental and basic activities of daily living (participation), self-reported diseases (health conditions), physical performance, common mental disorders, sleep quality and nutritional status (body functions and structures), medications (environmental factors), and self-esteem (personal factors) (Fig 1).

Physical activity and sedentary behavior were measured using the long form of the International Physical Activity Questionnaire (IPAQ), adapted for Brazilian older adults [16,17]. The IPAQ questionnaire estimates the moderate-vigorous physical activity (MVPA) in four domains (occupation, transportation, housework, and leisure time). A total physical activity score was calculated using the sum of the number of minutes of total moderate activity (including walking), plus two times the number of minutes of vigorous activity. Physical activity time was determined in minutes/week as the time spent in moderate to vigorous intensity activities.

For sedentary behavior, participants reported the time spent sitting on weekdays and weekend days. A weighted average [(week × 5) + (weekend × 2)]/7 was used to estimate time spent in sedentary behavior during a typical day.

Performance in basic activities of daily living (BADL) and instrumental activities of daily living (IADL) were assessed using the Katz Index [18] and the Lawton and Brody Scale [19], respectively. For data analysis, the total score on the scales was considered, and disability was determined by the highest BADL score and the lowest IADL score.

The number of self-reported diseases was determined from a list of diseases. Physical performance was measured according to the Fullerton battery of tests [20]. Common mental disorders were assessed by the Self-Reporting Questionnaire-20 [21], sleep quality was assessed by the Pittsburg Sleep Quality Index [22], and nutritional status was assessed by the Mini Nutritional Assessment [23]. The number of medications was reported by the older adults and self-esteem was measured by the Rosenberg Self-Esteem Scale [24]. Table 1 presents information on the variables and instruments used.

**Table 1:** Variables tested in the model.

Variable	Measuring instrument	Measure collection
Physical activity	Long form of the International Physical Activity Questionnaire, adapted for Brazilian older adults	Minutes/week as the time spent in moderate to vigorous intensity activities.
Sedentary behavior	Long form of the International Physical Activity Questionnaire, adapted for Brazilian older adults	Time spent sitting on weekdays and weekend days. A weighted average [(week × 5) + (weekend × 2)]/7 was used to estimate time spent in sedentary behavior during a typical day.
Basic activities of daily living	Katz Index	Investigates self-care activities like bathing, grooming, toileting, transferring, eating and continence. For data analysis, the total dependence score on the scale was considered, ranging from 0 (independent) to 12 (totally dependent).
Instrumental activities of daily living performance	Lawton and Brody Scale	Takes into account activities such as using the phone, going to distant places using some form of transport, shopping, preparing your own meal, cleaning and tidying the house, taking medication and dealing with finances. For data analysis, the total independence score on the scale was considered, ranging from 0 (totally dependent) to 14 (independent).
Upper limb flexibility	Fullerton battery of tests—The Back Scratch Test.	With one hand reaching over the shoulder and one up the middle of the back, the distance (cm) between extended middle fingers (+ or -) was recorded.
Lower limb flexibility	Fullerton battery of tests—The Chair Sit and Reach Test.	From a sitting position in front of a chair, with leg extended and hands reaching toward toes, the distance (cm) (+ or -) between extended fingers and tip of toe was recorded.
Upper limb strength and endurance	Fullerton battery of tests—The Biceps Curl Test.	This requires people to repeatedly lift a 5 lb (2.27 kg) weight (for women) or an 8 lb (3.63 kg) weight (for men) for 30 seconds. The number of lifts was recorded.
Lower limb strength and endurance	Fullerton battery of tests—The Chair Stand Test.	This requires people to repeatedly stand up from and sit down on a chair for 30 seconds. The number of stands was recorded.
Agility and balance	Fullerton battery of tests—8-Foot Up-and-Go	Number of seconds required to get up from a seated position, walk 8 feet (2.44m), turn, and return to seated position
Aerobic resistance	Fullerton battery of tests—2-Minute Step Test	Number of full steps completed in 2 minutes, raising each knee to a point midway between the patella (kneecap) and iliac crest (top hip bone). Score is number of times right knee reaches the required height.
Common mental disorders	Self-Reporting Questionnaire-20—SRQ-20	The SRQ-20 has 20 items on physical and psychic symptoms, with a range of dichotomous answers (yes or no) for the detection of common mental disorders. Each affirmative answer has a score of 1 for the final score from the sum of these values. The scores obtained are related to the probability of presence of non-psychotic disorder, ranging from 0 (no probability) to 20 (extreme probability).
Sleep quality	Pittsburg Sleep Quality Index	The PSQI is a 19-item self-report measure of sleep quality over the previous month. It consists of seven component scores, each rated on a 0 to 3 scale, with higher scores implying greater difficulties. These seven component scores can be summed to form a single global score, which ranges from 0 to 21, with higher scores reflecting greater overall sleep disturbance.
Nutritional status	Mini Nutritional Assessment (MNA)	The MNA is composed of four parts: (I) anthropometrical measurements; (II) global assessments; (III) dietary questionnaire; and (IV) subjective assessment. The total obtainable score is 30 points, with lower scores reflecting worse nutritional status.
Number of medicines	Self-reported	The number of medicines was assessed using the question: "How many medicines do you currently use?". The amount of drugs consumed by the older adults was considered.
Self-esteem	Rosenberg Self-Esteem Scale	To assess the individual’s perception of its own self-esteem. It is composed by 10 statements, answered in a 4-point Likert-type scale (1 = totally disagree to 4 = totally agree). The global score ranging from 10 (low self-esteem) to 40 (high self-esteem).
Self-reported diseases	ICD-10 List	The following were included: heart problems, high blood pressure, stroke, asthma/bronchitis, rheumatism/arthritis/arthrosis, spine/low back pain, osteoporosis, type 2 diabetes mellitus, cancer and kidney problems (kidney stones and urinary tract infection). The number of diseases presented by the older adults was considered.

The data were entered in double entry into Epidata software, version 3.1b. A Path Analysis was performed [25]. The model specified in the present study was based on the theoretical framework of the ICF and tested the associations between activity (exogenous variables) and direct and indirect participation (endogenous variables), mediated by the variables related to the health conditions, functions and structures of the body, environmental factors and personal factors.

The variables of the hypothetical initial model were previously assessed by bivariate models and Pearson correlation coefficients (p <0.05). The model estimation was performed using the maximum likelihood method; a normality analysis for endogenous variables was performed, and normality was verified with the asymmetry (Sk <3.0) and kurtosis (Ku <10.0) measurements [26]. Quality of fit was assessed by the following parameters: the chi-square test (x^2^), whose p value should be greater than 0.05 to indicate an adequate fit; root mean square error of approximation (RMSEA), whose p value should be less than or equal to 0.05 to indicate a proper fit; and the goodness of fit index (GFI), comparative fit index (CFI) and Tucker-Lewis index (TLI), whose p values should be 0.90 or above to indicate a proper fit.

Since the initial adjusted model was not considered appropriate to explain the correlational structure of the observed variables, the model was respecified; no significant pathways were removed, and modification indices were calculated [25]. The two models were compared by the chi-square difference test (x^2^).

The results were presented using standardized direct and indirect regression coefficients. Indirect standardized coefficients were obtained by multiplying the direct path coefficients between the variables. The indirect effect was considered significant when the trajectories between the mediators were significant [25]. The analysis was performed using the IBM SPSS statistics and IBM SPSS AMOS 24 (IBM SPSS Inc., Chicago, IL, 2016).

## Results

The participants in the study had a mean age of 69.86 ± 8.02 years; 61.8% (n = 259) were women, and 44.5% (n = 186) had ≥4 years of education, 55.1% (n = 231) lived with children or grandchildren, 48.9% (n = 205) were married and 44.2% (n = 185) belonged to economic classification classes D-E.

Table 2 presents the measures of central tendency and dispersion for the variables tested in the hypothetical model.

**Table 2:** Measures of central tendency and dispersion of the analyzed variables.

Variables	Mean (SD)
Moderate-vigorous physical activity (min/week)	389.0 (596.1)
Sedentary behavior (min/day)	423.5 (155.5)
Number of diseases	1.7 (1.3)
Upper limb flexibility (cm)	-9.6(10.5)
Lower limb flexibility (cm)	-3.1 (14.7)
Upper limb strength and endurance (repetitions)	18.4 (5.9)
Lower limb strength and endurance (repetitions)	14.2 (5.9)
Agility and balance (sec)	6.4 (3.5)
Aerobic resistance (steps)	74.6 (27.2)
Common mental disorder (0 to 20)	4.0 (3.8)
Sleep quality (0 to 21 scale)	4.9 (3.6)
Nutritional Status (0 to 30 scale)	24.5 (2.9)
Self-esteem (range 10 to 40)	33.4 (4.1)
Number of medicines	2.4 (2.1)
IADL (scale 0 to 14)	11.5 (2.7)
BADL (0 to 12 scale)	0.3 (0.6)

IADL: Instrumental activities of daily living; BADL: Basic activities of daily living; cm: Centimeters; SD: Standard deviation; min/week: Minutes/week; min/day: Minutes/day; sec: Seconds.

The correlations between the variables included in the model are described in Table 3. The BADL score showed a significant correlation with the variables analyzed in the present study. For IADL, a significant correlation was also found with the analyzed variables, except for the flexibility of the lower limbs.

**Table 3:** Correlation between the variables included in the initial model.

	BADL	IADL	MVPA	SB	DISEASE	ULF	LLF	ULSE	LLSE	AGE	AR	CMD	SQ	NS	SE	MED
BADL	1.0															
IADL	-0.4**	1.0														
MVPA	-0.2**	0.3**	1.0													
SB	0.2**	-0.3**	-0.2**	1.0												
DISEASE	0.2**	-0.2**	-0.0	0.2**	1.0											
ULF	-0.1**	0.2**	0.1**	-0.1	-0.1**	1.0										
LLF	-0.2**	0.0	0.0	-0.1**	-0.0	0.1**	1.0									
ULSE	-0.2**	0.4**	0.1**	-0.2**	-0.1*	0.2**	0.1	1.0								
LLSE	-0.2**	0.4**	0.2**	-0.2**	-0.2**	0.3**	0.2**	0.6**	1.0							
AGE	0.2**	-0.5**	-0.2**	0.3**	0.1*	-0.3**	-0.1	-0.5	-0.6**	1.0						
AR	-0.2**	0.4**	0.2**	-0.3**	-0.2**	0.2**	0.1	0.6**	0.5**	-0.6**	1.0					
CMD	0.2**	-0.2**	-0.1	0.0	0.4**	-0.0	-0.1	-0.1*	-0.1*	0.1*	-0.1*	1.0				
SQ	0.1**	-0.1**	-0.1	0.1*	0.3**	-0.0	-0.1	-0.1*	-0.1**	0.1	-0.1*	0.4**	1.0			
NS	-0.1**	0.3**	0.1	-0.1**	-0.3**	0.1	-0.0	0.2**	0.2**	-0.2**	0.2**	-0.4**	-0.3**	1.0		
SE	-0.2**	0.3**	0.3**	-0.1*	-0.1*	0.1	-0.0	0.1**	0.1**	-0.2**	0.1**	-0.3**	-0.1**	0.3**	1.0	
MED	0.1**	-0.2**	-0.1	0.2**	0.6**	-0.1**	-0.1*	-0.1**	-0.2**	0.2**	-0.2**	0.2**	0.2**	-0.2**	-0.1**	1.0

BADL: Basic activities of daily living; IADL: Instrumental activities of daily living; MVPA: Moderate-vigorous physical activity; SB: Sedentary behavior; ULF: Upper limb flexibility; LLF: Lower limb flexibility; ULSE: Upper limb strength and endurance; LLSE: Upper limb strength and endurance; AGE: Agility and balance; AR: Aerobic resistance; CMD: Common mental disorder. SQ: Sleep quality; NS: Nutritional status; SE: Self-esteem; MED: Medicines

*p < 0.05.

** p < 0.01.

**Fig 2:**
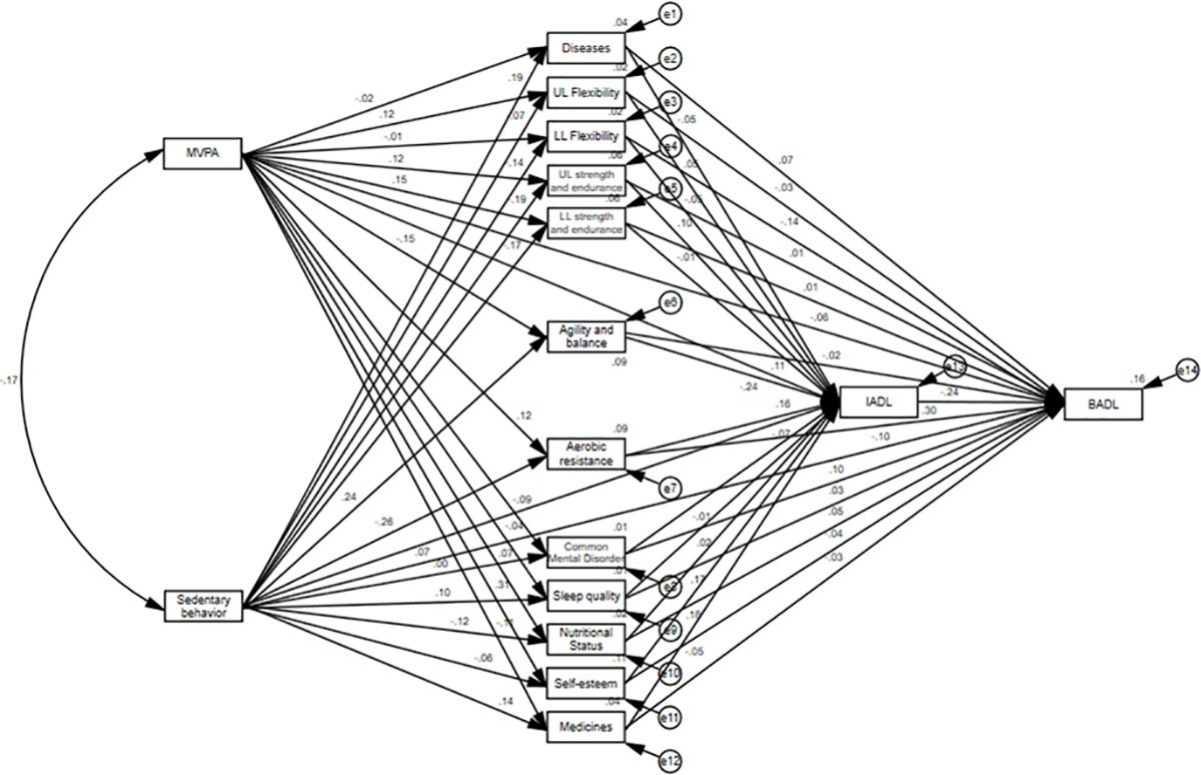
Initial model for explaining the effects of physical activity and sedentary behavior with ICF. MVPA: Moderate-vigorous physical activity; UL Flexibility: Upper limb flexibility; LL Flexibility: Lower limb flexibility; UL strength and endurance: Upper limb strength and endurance; LL strength and endurance: Upper limb strength and endurance; BADL: Basic activities of daily living; IADL: Instrumental activities of daily living.

In the initial model tested (Fig 2), the following adjustment quality indices were found: x2 (df) = 1283.53 (66), p <0.001; CFI = 0.293; GFI: 0.659; TLI: -0.286; and RMSEA: 0.211. These results indicated a poor quality of fit, and therefore, the model was respecified.

**Fig 3:**
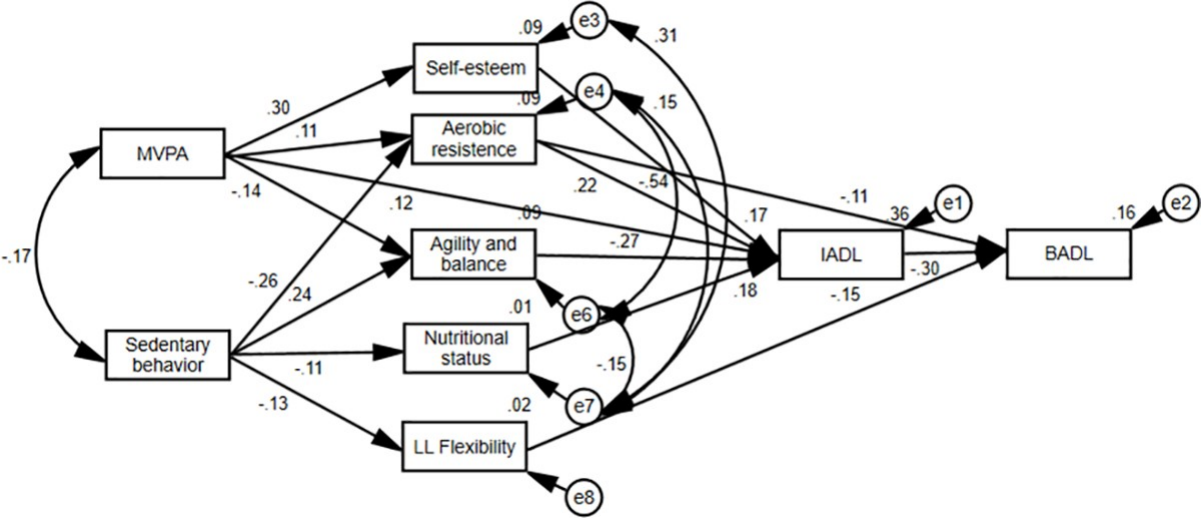
Final model explaining the effects of physical activity and sedentary behavior with ICF. MVPA: Moderate-vigorous physical activity; LL Flexibility: Lower limb flexibility; BADL: Basic activities of daily living; IADL: Instrumental activities of daily living.

After the respecification step, with the elimination of nonsignificant pathways and the inclusion of correlations between the errors of the mediating variables, the final model (Fig 3) showed good fit quality indices: x2 (df) = 21.23 (16), p = 0.08; CFI = 0.991; GFI: 0.989; TLI: 0.980; and RMSEA: 0.028.

Additionally, the respecified model demonstrated a significantly higher quality of fit than the initial model ([gl = 50] Δx2 = 1262.33; p <0.05). Overall, the final model explained 36% of the variability of disability in IADL and 16% of that in BADL.

Table 4 presents the standardized coefficients and their respective significance of the direct trajectories in the final model analyzed.

**Table 4:** Direct standardized coefficients for the variables analyzed in the final model.

Estimator	Effects	p
**IADL**		
MVPA	0.121	0.004
Self-esteem	0.174	< 0.001
Aerobic resistance	0.223	< 0.001
Agility and balance	-0.268	< 0.001
Nutritional status	0.183	< 0.001
**BADL**		
Aerobic resistance	-0.107	0.033
LL flexibility	-0.150	< 0.001
IADL	-0.303	< 0.001
**Self-esteem**		
MVPA	0.305	< 0.001
**Aerobic resistance**		
MVPA	0.109	0.019
Sedentary behavior	-0.261	< 0.001
**Agility and balance**		
MVPA	-0.135	0.004
Sedentary behavior	0.238	< 0.001
**Nutritional status**		
Sedentary behavior	-0.114	0.014
**LL flexibility**		
Sedentary behavior	-0.133	0.006

MVPA: Moderate-vigorous physical activity; LL Flexibility: Lower limb flexibility; BADL: Basic activities of daily living; IADL: Instrumental activities of daily living.

In the final model, physical activity had direct (β = 0.121) and indirect effects on IADL disability. The indirect effect was mediated by self-esteem (β = 0.053), aerobic endurance (β = 0.024) and agility and balance (β = 0.036). However, sedentary behavior had only an indirect effect on IADL, which was mediated by aerobic resistance (β = -0.058), agility and balance (β = -0.064) and nutritional status (β = -0.021).

Regarding disability in BADL, there were no direct effects of physical activity and sedentary behavior. Physical activity had an indirect effect mediated by IADL disability (β = -0.037) and aerobic endurance (β = 0.012). Sedentary behavior had an indirect effect mediated by aerobic resistance (β = 0.028) and flexibility of the lower limbs (β = 0.020).

## Discussion

The results of this study showed that IADL disability is directly associated with BADL disability. In addition, physical activity and sedentary behavior have a direct and indirect influence on disability in community-dwelling older adults.

Based on the ICF model, there may be an association between the activity domain (physical activity and sedentary behavior) and participation domain (BADL and IADL) that is mediated by the body functions and structures domain (nutritional status, agility and balance, aerobic endurance and flexibility of the lower limbs) and personal factors domain (self-esteem). The health condition (diseases) and environmental factor (medicines) domains did not influence the activity domain. Importantly, the health condition domain (diseases) did not remain in the final model. The theoretical models that were developed on disability prior to the ICF suggested that disability is a direct result of pathologies and body injuries within a biomedical discourse [27,28]. However, the present study indicated that health condition had no direct effect on BADL and IADL disability for the population studied. These results demonstrate that functionality is a biopsychosocial phenomenon and should be investigated independent of older adults’ health conditions [13].

The direct association of IADL disability with BADL can be explained by the hierarchy of activities, and IADL disability precedes the onset of BADL disability [29]. In other words, individuals with disability in BADL likely already show impairment in IADL, but not vice versa. Functionality in IADL involves multifaceted and cognitively challenging behaviors [30–32], while in BADL, functionality involves aspects related to functional mobility [33]. Therefore, it is very important that the modifiable factors for IADL in relatively functional older adults are identified to mediate subsequent BADL disabilities and/or other adverse outcomes [34,35].

Considering the final model of the present study, physical activity was directly associated with IADL disability, and the indirect association was mediated by self-esteem, aerobic endurance and agility and dynamic balance. Regarding BADL disability, only physical activity showed an indirection association that was mediated by aerobic resistance and IADL. Regular physical activity has been associated with IADL [36] and BADL in older adults [36,37]. Moreover, physical activity is considered a predictor of the absence of disability in IADL, being that, in 624 older adults, it was observed that 280 min/week (women) or 410 min/week (men) were the best cutoff values for predicting the absence of disability [38].

One of the indirect associations between physical activity and IADL was mediated by self-esteem. Self-esteem is considered a promoter of physical, psychological and social well-being, as it encourages participation in social and cognitive activities and promotes active aging [39]. Evidence shows that physical activity is directly associated with self-esteem [40] and has short-term and long-term effects on self-esteem [41,42]. A longitudinal study that assessed social activity and leisure with well-being among older adults found that more active older adults expressed higher self-esteem than did less active older adults [43]. In addition, physical activity programs have been used as a way to improve older adults’ self-esteem [44]. It is believed that physical activity increases the self-esteem of older adults by improving the perceptions of their own physical condition and associating them with a more attractive body [42], which influences their body image [45].

On the other hand, low self-esteem is associated with worse scores of social participation in the quality of life facet, which indicate low involvement in social activities [46]. In addition, another study showed that people with a negative mood live for on average 7.5 fewer years than those who are positive [47], and those with negative perspectives on aging have a slower recovery from disability [47].

The indirect association between physical activity and disability in IADL and BADL was also mediated by physical function. Muscle strength, aerobic endurance, flexibility, agility, and dynamic balance variables are known to decline with age [48–50], leading to an impaired ability to perform BADL [51] and IADL [52]. According to a meta-analysis, physical activity improves physical function in community-dwelling older people [53]. Other studies have also shown that the level of lifelong physical activity is associated with physical function [54,55]. It is believed that the relationship between activity and physical function may be bidirectional, i.e., increased physical activity may improve physical function, and higher levels of physical function may increase a person's willingness to be physically active [56]. Among the mediators of physical function, aerobic endurance was a significant mediator for the associations between physical activity and disability in IADL and between physical activity and disability in BADL.

In the general population, higher levels of cardiorespiratory fitness have been observed in individuals with the highest level of physical activity [57,58]. In older adults, aerobic resistance has been positively associated with moderate to vigorous physical activity levels [59]. It was also found that active older adults showed better performance in the aerobic fitness test than did inactive older adults [48]. In addition, a longitudinal study found that older women participating in a regular physical activity program had a smaller decline in aerobic fitness than their inactive peers [60]. Declines in aerobic fitness lead to a loss of independence in older adults [61]. Those who have reported having limitations when walking approximately 400 meters are at a higher risk for disability in BADL and IADL [62]. However, a longitudinal study (13 years) has shown that aerobic exercise protects older adults against disability and is associated with a long disability-free life [63].

Agility and balance also showed to be significant mediators between physical activity and disability in IADL. With aging, coordination and balance deteriorate, and this process is caused by several factors, such as somatosensory damage, vestibular problems, diseases, neuromuscular issues, and muscle weakness [64]. Studies show that a longer time to perform tests involving agility skills (speed and coordination) and dynamic balance (maintenance of postural stability during movement) may indicate mobility problems and a high risk for falls [65,66].

Considering that a fall is an event that directly affects the functionality of older adults, a longitudinal study conducted with older adults without disability found that those who suffered falls had greater difficulty in performing activities of daily living [67]. Additionally, another longitudinal study identified an association between a fear of falling and disability in older adults [68]. On the other hand, physical activity is associated with higher levels of physical function for balance and mobility in older adults [56]. A meta-analysis determined that free physical activity improves balance, particularly the type that is important for fall prevention, in community-dwelling older adults [69].

In the present study, sedentary behavior was not directly associated with either IADL or BADL disability. However, it was indirectly associated with IADL disability, and the association was mediated by aerobic endurance, agility and dynamic balance and nutritional status. It was also indirectly associated with BADL disability, and the association was mediated by aerobic resistance and flexibility of the lower limbs. Increased time spent in sedentary behavior has been shown to increase the risk for IADL and BADL disability [70]. In contrast, impaired sedentary behavior has been associated with improved IADL disability [11] in older adults. One of the indirect associations between sedentary behavior and IADL was mediated by nutritional status. Malnutrition is accompanied by the loss of body weight, muscle mass and strength and is considered an etiological factor for the development of sarcopenia [71].

Although it is evident that long-term exposure to sedentary behavior throughout life is associated with an unhealthy diet [72], the relationship between sedentary behavior and malnutrition is not yet clear. As previously verified in a meta-analysis, there are no studies that have evaluated the relationship between the time exposed to sedentary behavior and malnutrition in older adults [73]. However, a systematic review investigated the possible modifying determinants of malnutrition in older adults, and the level of physical activity, physical function and self-rated health were moderately related to the risk of malnutrition [74]. Thus, although the relationship between sedentary behavior and malnutrition is not clear, studies have shown a relationship between sedentary behavior and modifiable determinants of malnutrition in older adults that is attributable to reduced physical function [75] and a reduced self-perception of health [76].

The intake of dairy products, fruits and vegetables is known to be inversely associated with disability [77]. In addition, in a study in Brazilian older adults, low body weight was associated with disability in activities of daily living [78]. Thus, nutritional interventions can mediate weight loss and improve performance in activities of daily living in older adults at risk of malnutrition [79].

The other mediators between sedentary behavior and IADL and BADL disability were related to physical function. Evidence shows that sedentary behavior is associated with impaired physical function [54,59]. However, evidence also shows that breaks in sedentary behavior are associated with better physical function in older adults [80]. Aerobic resistance has been suggested to be a mediator between sedentary behavior and disability in IADL and between sedentary behavior and disability in BADL. In a population aged 40–75 years, it was found that more time spent in sedentary behavior was associated with a shorter distance covered in the 6-minute walk test [81]. In older adults, evidence also shows that sedentary behavior is associated with worse aerobic fitness scores [58]. Better cardiovascular health is a protective factor for poor physical function [82]. However, sedentary behavior has been associated with a risk of cardiovascular disease [83].

A longitudinal study analyzed the independent associations of 10 years of sedentary behavior changes, moderate to vigorous physical activity, and objective measurements of cardiorespiratory fitness with simultaneous changes in overall cardiometabolic risk and its individual components in 425 subjects (55.83 ± 9.40 years). Increased sedentary behavior was found to be associated with more detrimental changes in overall cardiometabolic risk, waist circumference, HDL cholesterol, and triglycerides, regardless of changes in moderate to vigorous physical activity [84]. Regarding disability, it has also been found that better cardiovascular health is associated with a lower risk of disability over 9 years in older adults [85]. In addition, lower levels of cardiorespiratory fitness in adult men increase the risk of disability requiring pension [86].

The association between sedentary behavior and disability in IADL was also mediated by agility and dynamic balance. Previous studies have shown that a longer time spent in sedentary behavior is associated with poorer performance regarding agility and dynamic balance [54,59]. The relationship between sedentary behavior and agility and dynamic balance can be explained by reduced muscle mass and mobility. In a study in older adults, it was shown that television viewing time is negatively associated with total body and leg lean mass [87]. Agility and dynamic balance are associated with mobility [88], which is considered an essential component for healthy aging and can predict disability [89]. In addition, sedentary behavior is associated with mobility in older adults. Liao et al. [90] found that in females aged 65–84, sedentary behavior was associated with the 5-meter walk test results, and those with a longer time spent in sedentary behavior had worse mobility. Additionally, it was also found that a longer time spent in sedentary behavior was not only associated with poorer walking performance but was also indicative of impaired static balance [91].

The association between sedentary behavior and BADL disability was mediated by the flexibility of the lower limbs. A study indicated that age-related loss of mobility is very joint-specific and may be related to distinct patterns of routine use of major joints throughout one’s life [92]. Regarding the lower limbs, it was found that hip joint mobility decreases by approximately six degrees per decade in individuals aged 55 to 86 years [93]. Evidence regarding flexibility is limited. Hip extension, dorsiflexion, and plantar ankle flexion range of motion are known to be important factors that influence physical function in community-dwelling older women [94]. In addition, it has been found that ADLs that require sitting and standing movements are partially dependent on flexibility [95]. This study indicates that the range of motion of the lower limbs is a predictor of disability in older adults [96]. Progressive reductions in range of motion are probably caused by the shortening of muscles or connective tissues due to reduced compliance of the joint structures and degenerative changes, as well as decreased muscle stretching, which results from decreases in daily physical activity with advancing age [97].

As seen in the present study, the health-focused approach to healthy aging should be multidimensional [98]. There is already evidence of interventions related to this result. A study found that encouragement for physical activity and proper nutrition are crucial for a well-targeted public health policy for healthy aging. In addition, psychosocial elements related to social participation, networking and life satisfaction are also considered beneficial for one’s health [98]. A recent study investigated the effect of a two-year multidimensional lifestyle intervention on the daily function of older people at risk for cognitive decline. The intervention included physical activity, nutritional counseling, vascular risk monitoring and management, cognitive training, and social activity. After two years, with the intervention, the level of daily function remained constant in the studied population [99]. Moreover, it is known that changes should be initiated during childhood, as it is considered a crucial period in life for the education on and promotion of healthy lifestyles that improve one’s health; the lifestyles should be maintained in adulthood to reduce risk for chronic diseases and disability later in life and counterbalance the emerging “pandemic” of inactive and sedentary lifestyles [100,101].

The limitations of the present study include the absence of a priori power analysis, the inability to determine the direction of causality, which is caused by the study design and the subjective measures used to assess physical activity and sedentary behavior. One strength of the study is the application of structural equation modeling analysis based on the ICF model, which allowed us to explain the association of physical activity and sedentary behavior with functionality.

## Conclusions

Physical activity was directly and indirectly associated with IADL, and the indirect association was mediated by self-esteem, aerobic endurance, agility and balance. The time of exposure to sedentary behavior was indirectly associated with IADL, and the association was mediated by aerobic resistance, nutritional status and agility and balance. The time spent in physical activities was indirectly associated with BADL, and the association was mediated by aerobic resistance and IADL. The association of sedentary behavior with BADL was mediated by aerobic resistance and lower limb flexibility.

The panorama presented in the present study points to a functionality model in which the impact of the lifestyle of the older adults in their activities of daily living occurs indirectly. It is believed that future investigations that reinforce this view driven by the biopsychosocial model, from a predominant biomedical point of view, can strengthen intervention strategies for the postponement of functional disability.

## Supporting information

S1 File (XLSX)
